# Directed Evolution and Engineering of Gallium-Binding Phage Clones—A Preliminary Study

**DOI:** 10.3390/biomimetics4020035

**Published:** 2019-05-08

**Authors:** Nora Schönberger, Christina Zeitler, Robert Braun, Franziska L. Lederer, Sabine Matys, Katrin Pollmann

**Affiliations:** 1 Helmholtz Institute Freiberg for Resource Technology, Helmholtz-Zentrum Dresden-Rossendorf, Bautzner Landstraße 400, 01328 Dresden, Germany; c.zeitler@hzdr.de (C.Z.); r.braun@hzdr.de (R.B.); f.lederer@hzdr.de (F.L.L.); s.matys@hzdr.de (S.M.); k.pollmann@hzdr.de (K.P.); 2 Institute of Nonferrous Metallurgy and Purest Materials, TU Bergakademie Freiberg, Leipziger Str. 34, 09599 Freiberg, Germany

**Keywords:** phage surface display, gallium, metal–peptide interaction, site-directed mutagenesis, cysteine, peptide structure

## Abstract

The phage surface display technology is a useful tool to screen and to extend the spectrum of metal-binding protein structures provided by nature. The directed evolution approach allows identifying specific peptide ligands for metals that are less abundant in the biosphere. Such peptides are attractive molecules in resource technology. For example, gallium-binding peptides could be applied to recover gallium from low concentrated industrial wastewater. In this study, we investigated the affinity and selectivity of five bacteriophage clones displaying different gallium-binding peptides towards gallium and arsenic in independent biosorption experiments. The displayed peptides were highly selective towards Ga^3+^ whereby long linear peptides showed a lower affinity and specificity than those with a more rigid structure. Cysteine scanning was performed to determine the relationship between secondary peptide structure and gallium sorption. By site-directed mutagenesis, the amino acids of a preselected peptide sequence are systematically replaced by cysteines. The resulting disulphide bridge considerably reduces the flexibility of linear peptides. Subsequent biosorption experiments carried out with the mutants obtained from cysteine scanning demonstrated, depending on the position of the cysteines in the peptide, either a considerable increase in the affinity of gallium compared to arsenic or an increase in the affinity for arsenic compared to gallium. This study shows the impressive effect on peptide–target interaction based on peptide structure and amino acid position and composition via the newly established systematic cysteine scanning approach.

## 1. Introduction

The interaction of biomolecules with metals is one of the most fascinating mechanisms in nature. The implementation of such mechanisms in technical applications is particularly promising and has been controversially debated in biomedicine [1], biotechnological production [2,3,4], and nanotechnology [5,6,7,8]. Their use for biotechnological applications in the resource technology [9] is considered to be particularly innovative. Highly specialized biomolecules are used for the selective biosorption of metals or metal-containing particles. In particular, naturally occurring metalloproteins are used, which can be recombinantly modified for technical usage [10]. 

A major disadvantage of larger protein structures is their low selectivity for certain materials or metal ions. Therefore, the use of less complex biomolecules has become attractive. Especially, siderophores and short peptides are promising candidates for biomining [9,10]. Actually, nature offers a wide range of such structures responsible for the recruitment of certain vital trace elements. Accordingly, naturally occurring biomolecules interact primarily with metals that are abundant in the biosphere [11]. Metals that occur rarely and are bound stable in ores do not play a role in the metabolism of most living organisms and therefore do not have naturally occurring ligands in the narrower sense.

For the production of highly specialized biomolecules that bind technological relevant metals, it is therefore important to find alternative approaches. One promising approach is the directed evolution of peptides. Inspired by natural evolution, which resulted in highly specialized biomolecules, the phage surface display technique systematically selects randomized peptide sequences for a certain target. This method has already been used to identify several peptides that interact specifically with metal-containing particles, surfaces, or metal ions. Such peptides are mainly exploited for the development of biocomposites and nanomaterials and have a pioneering position in molecular biomimetics [12]. However, several previous studies have shown that metal ion-binding peptides can also be used to differentiate between metals [13] and even between different oxidation states of a metal [14].

In this work, the biosorption of gallium and arsenic on peptides was investigated. Gallium is considered to be a high-tech metal. Due to its important role in the electronics industry, it is regarded as strategically critical [15]. Gallium is currently obtained mainly from primary raw material sources. The extraction from ores or minerals as a main product is not profitable. Therefore, it is primarily extracted as a by-product of aluminium during the processing of bauxite. This process is well established and highly efficient but entails a high energy and water consumption [16]. For these reasons, a rising awareness of the finiteness of primary raw materials sources and the use of secondary resources is recently discussed. In this context, metal-containing wastewaters are an attractive source. For example, residues from the semiconductor industry accumulate during the production of GaAs wafers. Besides valuable amounts of gallium, these residues also contain large amounts of arsenic and other components in lower concentrations. However, as low metal concentrations, a complex metal matrix, and a variable composition of other accompanying substances make the usage of such waters challenging for traditional metallurgic approaches, and a metal binding peptide might be a suitable tool for the selective recovery of valuable metals from such solutions. 

In an earlier study, we reported the identification of binding motifs of gallium binding bacteriophage clones obtained from a commercial random peptide library. For the respective clones, a maximum of 14-fold better gallium biosorption compared to wild-type phage binding was achieved [17]. 

In this work, the binding affinity of these clones for gallium compared to arsenic was investigated. The bacteriophage clones present gallium-binding peptide motifs that are 12 amino acids long and of linear structure. For a later application, it is important that the peptides displayed on the bacteriophage are able to differentiate between gallium and arsenic with high affinity. The investigations on the chelation of metals by organic molecular structures showed that a lower entropy in the molecular structure of the ligands led to a more stable complexation [18,19]. However, there was a possibility that the displayed dodecameric peptides could be too long and flexible to sorb the gallium ions constantly equally well and stably. 

In the present study, one selected phage clone was optimized for its interaction with gallium ions. Using systematically altered site-directed mutagenesis, each amino acid of the peptide was replaced at least once by a cysteine. In order to bring the functional groups involved in the interaction into closer contact for a collective complexation of the target metal ion, an additional amino acid was exchanged for cysteine at a distance of four amino acids. Cysteine scanning is very suitable for reducing the entropy of the peptides presented on the bacteriophage and systematically validating the position of the disulphide bridge in order to identify optimized metal-binding peptide sequences.

## 2. Materials and Methods

### 2.1. Handling of Phage Display Library Clones

The gallium-binding bacteriophage clones that were investigated here all originated from the commercial random peptide library Ph.D.-12 (Ph.D.™-12 Phage Display Peptide Library Kit, New England Biolabs GmbH, Frankfurt am Main, Germany) (see Table 1).

For the propagation of individual clones, the bacterial host strain *Escherichia coli* K12 ER2738 (*F’ proA+B+ lacIq Δ(lacZ)M15 zzf::Tn10(TetR9)/fhuA2 glnV Δ(lac-proAB) thi-110 Δ(hsdS-mcrB)5*) was used. Thirty milliliters of lysogeny broth (LB) medium (10 g/L tryptone, 5 g/L yeast extract, 5 g/L NaCl, pH 7.5) was inoculated with *E. coli* and cultivated to an optical density of ~ 0.02 (λ = 600) at 37 °C while shaking. The cultures were infected with 1.5∙10^3^ pfU of the respective phage clone and incubated at 30 °C with vigorous shaking overnight. Ten milliliters of the preculture was used to infect 100 mL LB medium. The propagation batch was incubated for additional 7 h at 30 °C while vigorous shaking. The phage particles were purified from culture supernatant as described elsewhere [20]. The purified phage particles were resuspended in 0.5 mL TBS (TRIS-buffered-saline with 50 mM TRIS-HCl, 150 mM NaCl, pH 7.5) and were diluted to a final concentration of 5∙10^8^ pp/µL.

The phage particle concentration was determined by spectrophotometric quantitation as described by Scott [21]. The concentration is calculated based on the absorption of purified phage particles at 269 nm and 320 nm. It is considered that the size of filamentous bacteriophages is proportional to the number of nucleotides in the phage genome and therefore correlates with the protein content of the phage particle. This corresponds to about six times the DNA content. Using a molar extinction coefficient for the capsid, the concentration of physical particles can be calculated. (see Equation 1).

(1) $$\mathrm{Phage}\;\mathrm{particles}\;\mathrm{per}\;\mathrm{microliter}\;=\frac{\left(A_{269\;\mathrm{nm}}-A_{320\;\mathrm{nm}}\right)*6\times 10^{16}}{\left[\mathrm{nucleotides}\;\mathrm{in}\;\mathrm{the}\;\mathrm{phage}\;\mathrm{genome}\right]}$$

A nucleotide number of 7270 bases was calculated in case of Ph.D.-12 library phage particles as well as mutated progeny of clone C3.129 and 7222 bases in case of control phage without peptide insert (M13 KE Wt).

### 2.2. Site-Directed Mutagenesis Experiments

The phage clone C3.129 was used as the template for site-directed mutagenesis experiments. Each amino acid of the displayed peptide sequence HTQHIQSDDHLA was replaced at least once by a cysteine. In order to allow the formation of disulphide bridges within the displayed peptide, an additional amino acid was exchanged with cysteine at intervals of four amino acids (see Table 2).

Replicative form (RF) phage DNA was isolated from infected bacterial host cells. For this purpose, 5 mL LB medium was inoculated with *E. coli* cells and infected with approximately 5∙10^10^ pfU of the C3.129 clone as described above. The propagation batch was incubated at 30 °C with vigorous shaking overnight. The preparation of the RF DNA was performed using the PureYield™ Plasmid Miniprep System (Promega, US) according to the manufacturer’s instructions. The purified DNA was dissolved in water and used as template for site-directed mutagenesis polymerase chain reaction (PCR). 

The Q5^®^ Site-Directed Mutagenesis Kit (New England Biolabs GmbH) was used according to the manufacturer’s instructions. Mutagenesis primer pairs were designed to anneal back-to-back at the template DNA. For this purpose, the free online software NEBaseChanger™ (New England Biolabs GmbH) was used (see Table 2). However, to generate the mutants M1, M2, and M7 (see Table 1), it was necessary to carry out the site-directed mutagenesis PCR under usage of the Phusion^®^ High-Fidelity PCR Kit (New England Biolabs GmbH). The cycling conditions have been selected with 30 s and 98 °C initial denaturation; 25 cycles of 10 s and 98 °C denaturation, 30 s annealing (temperature according to Table 1) and 225 s and 72 °C elongation and a final elongation of 120 s and 72 °C. The resulting PCR product was subjected to a reaction with the provided Kinase-Ligase-DpnI (KLD) enzyme mix to circularize the PCR product and remove template DNA. 

The resulting plasmids were used to generate the mutant phage particles (M1–M7). For this purpose, *E. coli* host cells were made chemically-competent [22] by washing a freshly grown bacterial culture (OD_600_ ~ 0.45) subsequently with ice-cold 0.1 M magnesium chloride solution, 0.1 M calcium chloride solution, and 0.1 M calcium chloride solution with 15% glycerol. The DNA was introduced into the cells by heat shock at 42 °C for 30 s. The transformed cells were cooled down on ice, mixed with ice-cold SOC medium (2% *w/v* tryptone, 0.5% *w/v* yeast extract, 10 mM NaCl, 2.5 mM KCl, 10 mM MgCl_2_, 10 mM MgSO_4_, 20 mM glucose) and incubated for 30 min at 37 °C. 

The resulting culture was mixed with liquid TOP-agarose (LB medium containing 7 g/L agarose) to embed the infected cells and transferred to an agar plate containing LB medium with 15 g/L agar, 0.05 mg/mL isopropyl-β-d-thiogalactoside (IPTG) and 0.04 mg/ml 5-bromo-4-chloro-3-indolyl-β-d-galactoside (Xgal). The plate was incubated overnight at 37 °C. Single transformants appeared as blue plaques on the plate. They were picked and transferred to individual tubes containing TBS and incubated at 4 °C overnight to allow the bacteriophage particles to diffuse out of the agar while preventing any undesirable further propagation of the phage by residual *E. coli* cells. Remaining agarose and cells were removed from the solution. The phage particle containing supernatant was analyzed for successful mutagenesis by Sanger sequencing (GATC Biotech AG, Konstanz, Germany) using the oligonucleotide primer 5’-CCCTCATAGTTAGCGTAACG-3’.

### 2.3. Biosorption Experiments

The interaction of all bacteriophage clones with free metal ions in aqueous solution was investigated in biosorption experiments. Pilot experiments with different solutions have shown that the biosorption of metal ions on bacteriophage particles can best be investigated in low concentrated millimolar metal salt solutions (result not shown). 

A total of six different gallium and/or arsenic containing solutions with a metal concentration of approximately 3 mM were prepared (see Table 3). Solutions were kept at pH 3.2 using a sodium acetate buffer or at pH 8.5 using a sodium phosphate buffer. 

In each individual experiment, 500 µl gallium solution was mixed with 5∙10^10^ phage particles and incubated overnight at 4 °C while shaking. Phage particles were precipitated together with bound metal ions by the addition of 100 µL ice-cold solution with 20% polyethylene glycol 8000 and 2.5 M NaCl. The supernatant was thoroughly removed and phage particles and metal ions were resuspended in 1 mL TBS. The metal content of the solution was determined by inductively coupled plasma mass spectrometry (ICP-MS). Each Ph.D.-12 library clone and each mutant was tested with all six metal solutions in 6-fold redundant experiments.

## 3. Results and Discussion

### 3.1. Experimental Context

Industrial wastewaters are an attractive secondary resource for high-tech metals. Such waste streams often have a complex composition with varying pH values. The mixtures are a challenge for any technology aiming at selective metal recovery. The use of conventional methodologies is not efficient and therefore unprofitable. Metal-selective peptides derived from natural models could provide a solution.

In our study, we intended the development of metal-selective peptides that can be used for selective Ga removal from low concentrated wastewater streams from the semiconductor industry. These waters are characterized by a complex and variable composition with varying pH values depending on the process stage from which they emerged. In addition to valuable amounts of gallium, the solutions always contain considerable amounts of arsenic. In addition to a variable matrix of other constituents, such solutions contain approximately 4 mg/L gallium and arsenic with a pH ranging from 3.0–4.2 or >8.0. 

Within the scope of the project, Ga-selective peptide motifs are selected using the phage surface display technology and used as functional compounds for the development of biosorptive composites. The present study focused on the investigation of the biosorption capacity of different peptide-presenting phage clones for dissolved gallium and arsenic. 

The composition and pH value of metal solutions has great influence on metal speciation. With increasing complexity, the determination of such species becomes more elaborate. For less characterized complex solutions, such as those resulting from semiconductor production, the speciation remains unknown. Therefore, the biosorption of gallium and arsenic in simplified model solutions was investigated. In order to match the pH range of real industrial wastewater, acetate-buffered solutions with a pH value of 3.2 and phosphate-buffered solutions with a pH value of 8.5 were applied.

Although it was not possible to determine the exact composition of the metal speciation in these synthetic solutions, justified assumptions can be made on the basis of speciation studies for gallium and arsenic in aqueous solution, that were reported elsewhere [23,24]. It can be expected that at pH 3.2, a certain amount of gallium will be present as Ga^3+^, but also as Ga(OH)_2_^+^ and Ga(OH)^2+^. At pH 8.5, a dominance of the complex Ga(OH)_4_^−^ can be expected. Arsenic predominates at pH 3.2 as arsenate ion in the compound H_2_AsO_4_^−^. At pH 8.5 mainly HAsO_4_^2−^ can be expected.

### 3.2. Original Phage Clone Characterization

Phage clones used in this study were obtained in experiments performed with the commercial Ph.D.-12 library (New England Biolabs GmbH) and used for further investigations. In total, five different gallium-binding peptides were identified (referred to Table 1).

In order to determine the sorptive behavior of those five phage clones, six different model solutions at pH 3.2 or 8.5 were used. Each solution contained approximately 3 mM gallium or arsenic or 3 mM gallium and arsenic in combined solutions (see Table 3). A defined amount of phage particles was incubated in the model solutions. Phage particles were coprecipitated with bound metal, thus determining the interaction of bacteriophage clones with metal ions in solution. In addition to the gallium-binding bacteriophage clones, a clone corresponding to the wild-type (Wt), i.e., which did not present a peptide sequence, was also carried as a control experiment. This reference shows the influence of the peptide sequences of the clones.

**Figure 1:**
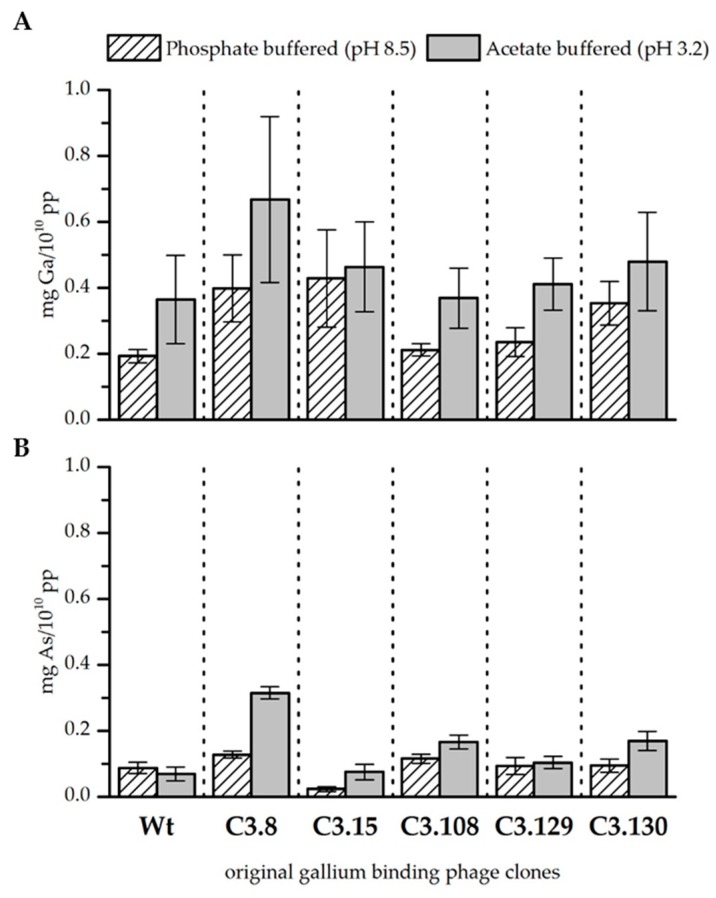
Biosorption of gallium (**A**) and arsenic (**B**) to original gallium-binding phage clones and wild-type phage control at pH 8.5 in phosphate buffered solution and pH 3.2 in acetate buffered solution. Results are expressed as the amount of sorbed gallium (mg) to 10^10^ phage particles (pp). Error bars represent the standard error.

**Figure 2:**
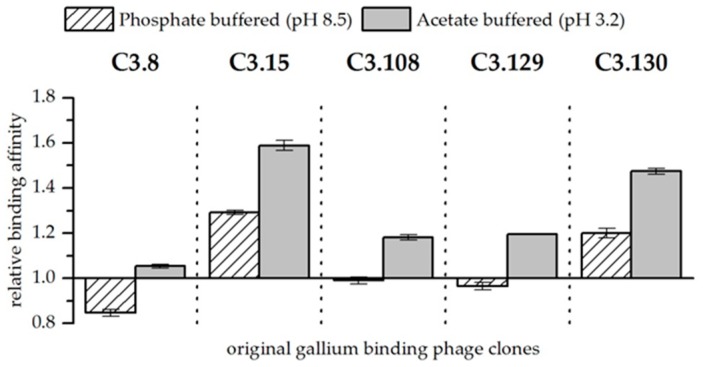
Relative binding affinity of original gallium-binding phage clones for gallium compared to arsenic at pH 8.5 in phosphate buffered solution and at pH 3.2 in acetate buffered solution. Results are expressed as the fold increase above the biosorption of wild-type bacteriophage particles. The values were normalized to the wild-type phage affinity for gallium compared to arsenic (Wt = 1). Error bars represent the standard error.

The precipitation experiments showed a great influence of the pH on the biosorption efficiency for both gallium and arsenic, as well as on the binding affinity of the bacteriophage clones for gallium in comparison to arsenic (see Figure 1 and Figure 2, as well as Table 4 and Table 5).

In general, gallium was bound better by the bacteriophage at pH 3.2 than at pH 8.5 when it was solely present in the solution. The wild-type phage control indicates that especially at lower pH, unspecific binding of gallium to the phage capsid takes place. However, the binding of gallium that occurs through the displayed peptides of the gallium binding phage clones is higher at pH 8.5 than at pH 3.2 (see Fig. 1A). In summary, it was found that clones C3.8 (TMHHAAIAHPPH), C3.15 (NYLPHQSSSPSR), and C3.130 (NYLPHQSSSPSR) bound gallium at pH 8.5 considerably better than the wild-type control. Since the clones differ from the wild-type only in the displayed peptide sequence, this result indicates an interaction between the peptide and gallium. The displayed peptides differ strongly in their amino acid composition and their arrangement so that no uniform binding domain or mechanism could be identified. However, the investigation of different chemical gallium ligands indicates that gallium is preferentially complexed in hexadentate structures by nitrogen and oxygen [25,26]. In principle, all five sequences meet such functional requirements for the complexation of gallium on the phage particle. At pH 3.2, gallium is partial present as Ga^3+^. Thus, it acts as a hard Lewis acid and can form complexes with the nitrogen and oxygen atoms of the displayed peptides. At pH 8.5, gallium occurs as Ga(OH)_4_^-^. The stability constant of this complex is very low [27]. It can, therefore, be assumed that single hydroxide ligands might be substituted in favor of building a more stable complex. Depending on the affinity of the displayed peptides for gallium, this might be the case. However, further studies of gallium–peptide interactions have to be done to resolve the binding mechanism. Nevertheless, there are indicated differences in the biosorption performance of the individual displayed peptides. Phage clones C3.8 (TMHHAAIAHPPH), C3.15 (NYLPHQSSSPSR), and C3.130 (NDLQRHRLTAGP) showed the highest biosorption of gallium at both pH values. These sequences differ mainly by the presence of large hydrophobic amino acids. The secondary structure of these peptides is characterized in particular by the inflexible and structure-giving character of proline. The phage clones C3.108 (SQALSTSRQDLR) and C3.129 (HTQHIQSDDHLA) showed lower biosorption for gallium. The higher flexibility of these peptides due to the absence of secondary structure stiffening amino acids in the peptide might be a reason for this [18].

Arsenic, if present solely in the solution, was sorbed by the bacteriophage as well. However, less arsenic was bound than gallium, although the concentration of the two elements in all solutions was similar. It is noticeable that the wild-type control sorbed arsenic slightly better at pH 8.5. The opposite is true for the gallium-binding clones tested. In particular, the clones C3.8 (TMHHAAIAHPPH) and C3.108 (SQALSTSRQDLR) bound noticeable more arsenic at both pH values than the wild-type control. Clone C3.8 stands out with its remarkable binding of arsenic, that is four times more than the control phage at pH 3.2. Clone C3.15 (NYPLHQSSSPSR) is noteworthy because it sorbs very little arsenic at both pH values. Arsenic is present at pH 3.2 and 8.5 as arsenate in the compounds H_2_AsO_4_^−^ and HAsO_4_^2−^ respectively. The coordination sphere of arsenic is covalently saturated in these compounds. This makes direct complexation by the peptides unlikely. It is therefore assumed that the interaction of bacteriophage clones is mainly based on electrostatic interactions with protonated groups. This assumption is supported by the fact that arsenic preferably interacts with those displayed peptides that have a higher isoelectric point than those that have a lower one (see Table 1).

When both elements are present in the solutions, they compete for sorption to the bacteriophage. Biosorption experiments with solutions at pH 3.2 and 8.5 and approximately equimolar concentrations of arsenic and gallium showed differences in their affinity for the respective elements. These results are of particular interest because gallium-binding peptides should be able to distinguish between gallium and arsenic for later application. In order to better assess the effect of the peptides presented on the bacteriophage, the wild-type phage was again used as a reference. Figure 2 shows the affinity of gallium-binding clones for gallium in comparison to arsenic and in relation to the biosorption to the wild-type control. In general, it was found that at pH 3.2, the peptides have a higher affinity for gallium compared to arsenic than at pH 8.5. Furthermore, at pH 3.2, all displayed peptides show a preference for interacting with gallium compared to arsenic. As already discussed, the Ga^3+^ ion occurring at low pH has a considerable advantage for the formation of a complex compared to arsenate. It is therefore assumed that the greater affinity is the result of chelating effects. At pH 8.5, only the phage clones expressing the peptides C3.15 (NYLPHQSSSPSR) and C3.130 (NDLQRHRLTAGP) showed a considerable better biosorption of gallium compared to arsenic (see Figure 2). In the case of the other peptides, even a slightly increased affinity for arsenic could be observed. Therefore, it is unlikely that these peptides are selectively binding gallium. It is assumed that the compact molecular structure makes direct binding of gallium difficult. However, substitutions of hydroxide ligands by peptides that bind gallium with a very high affinity is possible due to ligand substitution effects. 

### 3.3. Site-Directed Mutagenesis Experiments

The experiments with original library phage clones indicated that there is a relationship between selectivity and metal affinity and the secondary structure of the represented peptides. Long, flexible amino acid chains show poorer biosorption performances and lower specificity for gallium recognition. The fact that cyclic ligands can often form more stable complexes than open structures is known as the macrocyclic effect [28]. Accordingly, the biosorption of such peptides might be optimized by providing a more rigid secondary structure. In order to test this hypothesis, a peptide motif was specifically modified which, due to its flexible structure, was suspected of being less able to sorb gallium.

It has already been reported elsewhere that the targeted exchange of individual amino acids in a peptide by alanine can lead to considerable improvements [29] or deteriorations [30] in the functionality of the peptide. Alanine scanning enables the systematic replacement of amino acids in a peptide sequence. Alanine is usually used as a substitute because its methyl group is inert and is therefore excellent for testing the functionality of individual amino acids in a peptide. 

However, in this work, an alternative approach was chosen. The influence of structural changes on the selective recognition of metal ions of one gallium-binding peptide sequence was investigated. For this purpose, two amino acids with a distance of four amino acids were systematically replaced by cysteine (see Table 1). It was examined whether the systematic introduction of disulphide bridges, that would have a major influence on the secondary structure of the peptides and thus their flexibility, leads to a change in the biosorption of gallium and arsenic and whether an optimization of the sequence can perhaps be achieved by the changes. The clone C3.129 (HTQHIQSDDHLA) was chosen for cysteine scanning. The peptide sequence presented on the clone promised interesting possibilities for interaction with gallium, and in previous studies [17], a high affinity of the clone for an immobilized gallium target could be demonstrated. Nevertheless, only a low biosorption of free gallium ions could be observed in this work. Therefore, it was assumed that a more rigid secondary structure could have a beneficial effect on the complexation of gallium. All seven derivates of C3.129 were obtained by site-directed mutagenesis. The mutant phage particles display modified peptide sequences that are circularized due to cysteine-derived disulphide bridges.

### 3.4. Mutant Phage Clone Characterization

**Figure 3:**
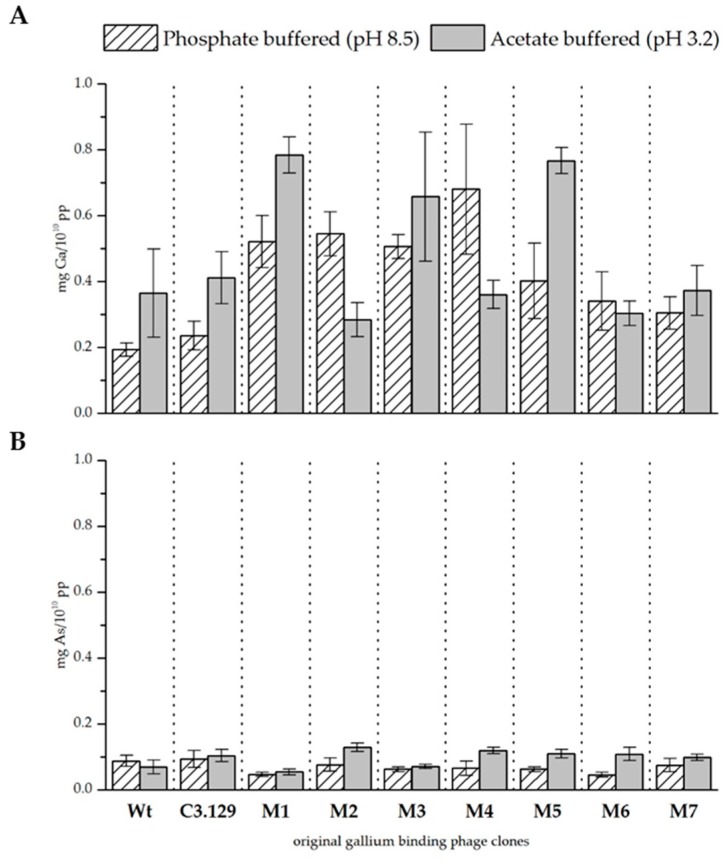
(**A**) Biosorption of gallium and (**B**) arsenic to mutant gallium-binding phage clones, original clone C3.129, and wild-type phage control at pH 8.5 in phosphate buffered solution and pH 3.2 in acetate buffered solution. Results are expressed as the amount of sorbed gallium (mg) to 10^10^ phage particles (pp).

The precipitation experiments were repeated for all seven mutants of the C3.129 clone. Figure 3 and Table 5 show the biosorption of gallium and arsenic at both pH values. The biosorption of gallium at pH 8.5 has been notably improved by cysteine scanning, not only with respect to the wild-type control phage, but also with respect to the original phage clone C3.129. The results for pH 3.2 indicate diverse effects. Major improvements were achieved by the changes in the mutants M1, M3, and M5. It is assumed that especially with the displayed peptides of these mutants, the improved gallium complexation might be achieved by macrocyclic effects. The biosorption of gallium by the mutant M2 has considerably decreased. The substitution of the hydroxyl amino acids at positions 2 and 7 in the mutant M2 resulted in a remarkable reduced biosorption of gallium. This indicates that the hydroxyl side chain functionality of serine and threonine at these positions is decisive for the complexation of Ga^3+^. 

The biosorption of arsenic at pH 3.2 could be increased by cysteine scanning of all mutants. A possible reason for this effect could be the very good interaction between arsenic and cysteines [31]. At pH 8.5, the sorption performance for arsenic was rather reduced. It is assumed that the more complex secondary structure restricted the possibilities for deposition on the peptide surface via electrostatic interactions.

**Figure 4:**
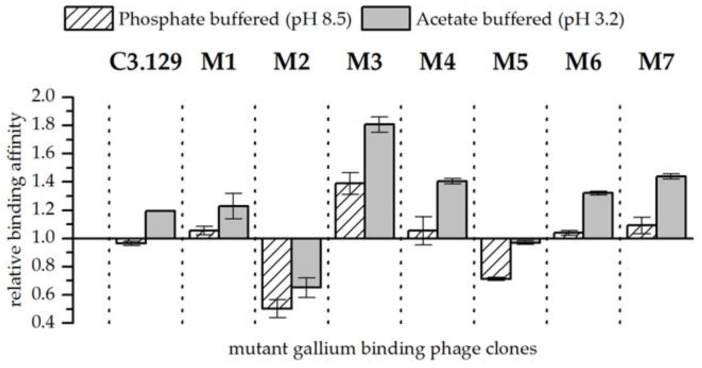
Relative binding affinity of mutant gallium-binding phage clones for gallium compared to arsenic at pH 8.5 in phosphate buffered solution and at pH 3.2 in acetate buffered solution. Results are expressed as the fold increase above the biosorption of wild-type bacteriophage particles. The values were normalized to the wild-type phage affinity for gallium compared to arsenic (Wt = 1). Error bars represent the standard error.

In experiments in which gallium and arsenic were present together in the solution, the affinity of bacteriophage for gallium compared to arsenic could be increased by the cysteine scanning (see Figure 4). Exceptions are the mutants M2 and M5. The respective changes in the amino acids increased the affinity of the mutants for arsenic compared to gallium. A highly increased affinity for gallium with arsenic at both pH points could be observed in the biosorption behavior of mutant M3. It can be speculated that by closing the disulphide bridge between the amino acids at positions 3 and 8, a pocket is created which is able to complex Ga^3+^ with high stability. However, more sophisticated structural analyses of synthetic peptides would be necessary to verify such an assumption. In addition to the strongly enhanced biosorption of gallium compared to arsenic from mutant M3, an improvement in gallium affinity at pH 3.2 for mutants M4 and M7 could also be observed. Interestingly, the mutants M3 and M4 each substituted aspartic acid once, and mutant M7 concealed both aspartic acid groups probably by the formation of a peptide ring that is displayed on the bacteriophage. Possibly, the acidic side chain functionality impeded the specific recognition of Ga^3+^.

## 4. Conclusions

In this study, the biosorption of dissolved gallium and arsenic by gallium-binding bacteriophage clones was investigated. A large influence of the pH value on the respective interactions could be determined. Many of the identified bacteriophage clones actually present peptide sequences that can sorb gallium at pH 3.2 and pH 8.5.

Experiments with arsenic have shown that arsenate ions were less well bound due to its chemical properties. In solutions containing both elements gallium and arsenic could be shown, that gallium might be complexed due to chelate effects and that the stable coordination sphere of arsenate allow only electrostatic interactions.

Since structural effects of the peptides were suspected to be involved in the specific binding of gallium, one of the gallium-binding peptide sequences was specifically modified. Site-directed mutagenesis experiments were performed to systematically replace amino acids with a cysteine pair. The modified peptides were presented on the bacteriophage. Biosorption experiments with the resulting bacteriophage mutants showed that the formation of a macrocyclic peptide structure has positive effects on the complexation of gallium. Furthermore, amino acids with hydroxyl side chain functionality were shown to be particularly important for the binding of gallium. 

We conclude that phage surface display is a suitable tool for the identification of metal-binding peptide sequences. By applying the tool to less abundant metals, the spectrum of naturally occurring metalloproteins could be extended. Furthermore, cysteine scanning has proven to be a very helpful complement for the engineering of peptides, that were obtained by directed evolution. It could be applied for the closer characterization of the structure-related interactions between peptide sequences and gallium. 

However, the results reported here primarily show the behavior of different bacteriophage clones that display peptides. Bacteriophage particles are practically not applicable. The production is problematic due to a high mutation rate of the phage and the large-scale industrial application of genetically modified organisms in wastewater treatment is not accepted. Since it is not possible to predict whether the peptides will behave exactly as they do on bacteriophages, experiments will now be conducted to characterize synthetic peptides. Suitable sequences will be immobilized on a suitable carrier and used for the recovery of gallium from industrial wastewater.

## Figures and Tables

**Table 1:** Bacteriophage clones used in this study: their name, origin, displayed peptide sequence, as well as the theoretical isoelectric point of the corresponding peptides.

Name	Sequence	pI	Origin
C3.8	TMHHAAIAHPPH	6.82	[17]
C3.15	NYPLHQSSSPSR	5.08	
C3.108	SQALSTSRQDLR	9.31	
C3.129	HTQHIQSDDHLA	5.70	
C3.130	NDLQRHRLTAP	9.61	
M1: H_1_C_1_/Q_6_C_2_	CTQHICSDDHLA	5.05	This work; SDM experiment of clone C3.129
M2: T_2_C_1_/S_7_C_2_	HCQHIQCDDHLA	5.70	
M3: Q_3_C_1_/D_8_C_2_	HTCHIQSCDHLA	6.25	
M4: H_4_C_1_/D_9_C_2_	HTQCIQSDCHLA	5.97	
M5: I_5_C_1_/H_10_C_2_	HTQHCQSDDCLA	5.05	
M6: Q_6_C_1_/L_1_1C_2_	HTQHICSDDHCA	5.70	
M7: S_7_C_1_/A_12_C_2_	HTQHIQCDDHLC	5.70	

pI: isoelectric point, C: clone, M: mutant, SDM: side-directed mutagenesis.

**Table 2:** Primer design for site-directed mutagenesis screening for clone C3.129 (HTQHIQSDDHLA).

Clone	T_A_	Pol	SDM Primer_Forward (5’-3’) & SDM Primer_Reverse (5’-3’)
M1	59 °C	Ph	CATATTTGTAGTGATGATCATCTTGCG/ CTGCGTACAAGAGTGAGAATAGAAAGGTAC
M2	59 °C	Ph	ATTCAGTGTGATGATCATCTTGCGGGTG/ATGCTGACAATGAGAGTGAGAATAGAAAGG
M3	61 °C	Q5	CAGAGTTGTGATCATCTTGCGGGTGGA/ AATATGACACGTATGAGAGTGAGAATAGAAAG
M4	61 °C	Q5	AGTGATTGTCATCTTGCGGGTGGAGGT/CTGAATACACTGCGTATGAGAGTGAGAATAG
M5	64 °C	Q5	GATGATTGTCTTGCGGGTGGAGGTTCG/ACTCTGACAATGCTGCGTATGAGAGTGAG
M6	65 °C	Q5	GATCATTGTGCGGGTGGAGGTTCGGCC/ATCACTACAAATATGCTGCGTATGAGAGTGAGAATAGAAAGGTAC
M7	62 °C	Ph	CATCTTTGTGGTGGAGGTTCGGCCGAA/ATCATCACACTGAATATGCTGCGTATGAGAGTG

T_A_: annealing temperature, Pol: DNA polymerase.

**Table 3:** Composition of gallium and arsenic-containing buffer solutions.

Metal Concentration	Concentration of Buffer Components	pH
2.8 mM Ga	0.0947 M Na_2_HPO_4_ 0.0053 M NaH_2_PO_4_	8.5
2.7 mM Ga	0.0995 M CH_3_COOH 0.0005 M NaCH_3_COO	3.2
3.1 mM As	0.0947 M Na_2_HPO_4_ 0.0053 M NaH_2_PO_4_	8.5
2.6 mM As	0.0995 M CH_3_COOH 0.0005 M NaCH_3_COO	3.2
1.2 mM Ga 1.3 mM As	0.0947 M Na_2_HPO_4_ 0.0053 M NaH_2_PO_4_	8.5
1.5 mM Ga 1.4 mM As	0.0995 M CH_3_COOH 0.0005 M NaCH_3_COO	3.2

**Table 4:** Relative biosorption of the clones increased above wild-type control. The values were normalized to the wild-type biosorption of gallium or arsenic (Wt = 1).

Clone	Relative Biosorption of Ga (Increase above Wt)				Relative Biosorption of As (Increase above Wt)			
	Phosphate Buffered (pH 8.5)		Acetate Buffered (pH 3.2)		Phosphate Buffered (pH 8.5)		Acetate Buffered (pH 3.2)	
C3.8	2.06 ±	0.52	1.83 ±	0.69	1.46 ±	0.12	4.58 ±	0.27
C3.15	2.21 ±	0.76	1.27 ±	0.37	0.27 ±	0.08	1.09 ±	0.34
C3.108	1.10 ±	0.09	1.01 ±	0.25	1.32 ±	0.15	2.41 ±	0.3
C3.129	1.22 ±	0.22	1.13 ±	0.22	1.06 ±	0.3	1.51 ±	0.28
C3.130	1.83 ±	0.34	1.31 ±	0.41	1.08 ±	0.23	2.46 ±	0.43

**Table 5:** Relative biosorption of the clones increased above wild-type control. The values were normalized to the wild-type biosorption of gallium or arsenic (Wt = 1).

Clone	Relative Biosorption of Ga (Increase above Wt)				Relative Biosorption of As (Increase above Wt)			
	Phosphate Buffered (pH 8.5)		Acetate Buffered (pH 3.2)		Phosphate Buffered (pH 8.5)		Acetate Buffered (pH 3.2)	
C3.129	1.22 ±	0.22	1.13 ±	0.22	1.06 ±	0.3	1.51 ±	0.28
M1	2.69 ±	0.41	2.15 ±	0.15	0.53 ±	0.07	0.78 ±	0.13
M2	2.81 ±	0.34	0.78 ±	0.14	0.87 ±	0.24	1.87 ±	0.19
M3	2.62 ±	0.19	1.80 ±	0.54	0.71 ±	0.08	1.04 ±	0.09
M4	3.52 ±	1.02	0.99 ±	0.12	0.74 ±	0.25	1.74 ±	0.13
M5	2.08 ±	0.59	2.10 ±	0.11	0.71 ±	0.08	1.59 ±	0.18
M6	1.76 ±	0.46	0.83 ±	0.1	0.52 ±	0.07	1.57 ±	0.29
M7	1.58 ±	0.25	1.02 ±	0.21	0.85 ±	0.23	1.43 ±	0.14
